# Novel Cryo-EM structures of the D1 dopamine receptor unlock its therapeutic potential

**DOI:** 10.1038/s41392-021-00630-3

**Published:** 2021-05-22

**Authors:** David R. Sibley, Kathryn D. Luderman, R. Benjamin Free, Lei Shi

**Affiliations:** 1 Molecular Neuropharmacology Section, National Institute of Neurological Disorders and Stroke, Intramural Research Program, National Institutes of Health, Bethesda, MD USA; 2 Computational Chemistry and Molecular Biophysics Section, Molecular Targets and Medications Discovery Branch, National Institute on Drug Abuse, Intramural Research Program, National Institutes of Health, Baltimore, MD USA

**Keywords:** Structural biology, Molecular neuroscience

Three recent articles published in *Cell*^1,2^ *and Cell Research*^3^ have reported multiple cryo-electron microscopy (cryo-EM) structures of the D1 dopamine receptor (DRD1) bound to either dopamine, various DRD1 agonists, or a positive allosteric modulator (PAM), while in complex with the active heterotrimeric G_s_ protein. These studies describe for the first time active-state structures of the DRD1, an exciting advancement in the field that will allow for a better understanding of selective agonist binding, DRD1 activation, G protein selectivity, and the provision of multiple templates to facilitate future drug design and discovery for this important therapeutic target.

Dopamine receptors are comprised of five subtypes subdivided into two subfamilies, D1-like (DRD1 and DRD5) and D2-like (DRD2, DRD3, and DRD4), that mediate diverse physiological effects, including the regulation of mood, reward, movement, and cognition. The DRD1 is the most abundant dopamine receptor and is highly expressed in the central nervous system (CNS). Not surprisingly, the DRD1 provides an attractive drug target for the treatment of numerous CNS disorders, including many that are associated with cognitive decline. However, this receptor has proven an elusive target, primarily due to liabilities inherent in existing DRD1 agonists, especially those based on the catechol scaffold that targets the dopamine binding (orthosteric) site. These compounds suffer from numerous drawbacks, including poor CNS penetrance, rapid metabolism, tolerance, and therapeutically limiting side effects. Various strategies have been used to address these limitations including the development of non-catechol agonists, signaling-biased compounds, and positive allosteric modulators (PAMs), which target receptor binding pockets distinct from the orthosteric site.^4^ However, high-resolution structural information has hitherto been unavailable severely limiting the potential for rational drug design and discovery.

**Fig. 1:**
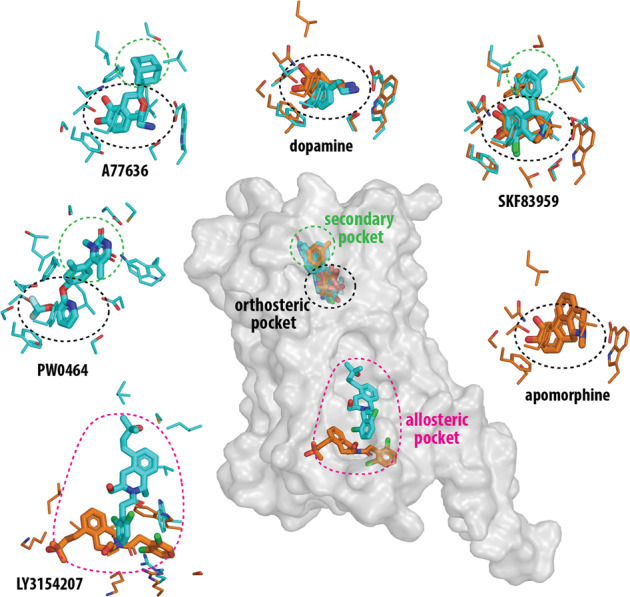
The DRD1 structures reveal orthosteric, secondary, and allosteric binding pockets. The relative locations of these pockets are indicated by colored ellipses in the central panel in which the active conformation of the DRD1 is shown in a transparent surface representation. The binding poses of selected ligands and their corresponding interacting residues (within 3.8 Å of the ligand) are shown in surrounding panels as labeled. The binding pockets are indicated by the ellipses in the same colors as the central panel. Those from Xiao, et al.^1^ are colored in cyan, those from Zhuang, et al.^2,3^ are colored in orange. In particular, three pairs of structures bound with dopamine, SKF83959, or LY3154207 from both groups are superimposed to demonstrate their divergences

Xiao et al. reported five structures of the DRD1-G_s_ complex bound to several distinct DRD1 agonists^1^ (Fig. 1). These included three catechol-containing agonists: A77636 (a full agonist), the antihypertensive drug fenoldopam (a partial agonist), and SKF83959 (a G-protein-biased agonist), as well as the non-catechol agonist, PW0464. In addition, this group described a DRD1-G_s_ complex simultaneously bound to dopamine in the orthosteric site and the PAM LY3154207 in an intracellular allosteric pocket. Based on the cryo-EM structures and molecular docking, the orthosteric site was described as being comprised of residues from transmembrane segments (TMs) 3, 5, 6, and 7 and capped by residues from extracellular loop 2 (ECL2). The catechol-containing agonists, including dopamine, interacted with several polar residues, including D103^3.32^, S198^5.42^, and S202^5.46^, as well as a network of nonpolar residues. A77636, fenoldopam, SKF83959, and PW0464 made further contact with an extended binding pocket comprised of residues from ECL2, and the extracellular regions of TMs 2, 3, 6, and 7 that may confer DRD1 selectivity to these agonists. The polar interactions between catechol-based agonists and TM5 serines including S198^5.42^, S199^5.43^, and S202^5.46^ are believed to function as microswitches that are essential for receptor activation. Notably, while exhibiting a different binding mode as the catechol agonists, the non-catechol agonist PW0464 still forms bonds with S198^5.42^ and S202^5.46^ via its fluorine atom. The PAM, LY3154207, was localized to a membrane-embedded binding site formed mainly by hydrophobic residues from intracellular loop 2 (ICL2), TM3, and TM4. Notably, the EM density of this site was not sufficiently detailed to unambiguously assign exact atom positions to the bound ligand; however, the authors presented mutagenesis and modeling data supporting a pose similar to one previously suggested for this ligand.^1,4^ Interestingly, the LY3154207 binding site is similarly located as an allosteric site identified in the β_2_-adrenergic receptor for a PAM named Cmpd-6FA.^5^

Zhuang, et al.^2^ simultaneously reported three cryo-EM structures of a DRD1-G_s_ complex bound to three catechol-containing agonists including the benzazepines SKF81297 (a full agonist) and SKF83959, as well as the pan dopamine receptor agonist apomorphine, which is used to treat Parkinson’s disease^2^ (Fig. 1). In addition, they reported a 2.8 Å resolution structure of a DRD2-G_i_ complex bound to bromocriptine, an ergot alkaloid that is a DRD2-preferring agonist and also an anti-parkinsonian drug. Comparison of this DRD2 structure with the DRD1 structures highlighted important differences in G-protein specificity, i.e., a more tilted TM6, longer TM5, and larger receptor-G protein interface in the DRD1-G_s_ complex. The DRD1 orthosteric binding pocket was similar to that defined by Xiao, et al.^1^, including several polar interaction motifs; however, there were notable differences in the binding pose and receptor interactions of SKF83959 (Fig. 1).^1,2^ Interestingly, from comparing the poses of the structurally related agonists SKF81297 (non-biased) and SKF83959 (G-protein-biased), it was suggested that steric packing between the methyl group in the azepine ring of SFK83959 and residues in TM7 may explain its signaling bias;^2^ however, a complete understanding of this mechanism will likely require the structural elucidation of a DRD1-β-arrestin complex.

A third report, from Zhuang, et al., described two cryo-EM structures of a DRD1-G_s_ complex simultaneously bound to the PAM, LY3154207, and either dopamine or the full agonist SKF81297^3^ (Fig. 1). In both structures, LY3154207 occupies an intracellular binding site similarly located to that described by Xiao, et al.^1^, however, the contact residues and LY3154207 pose differ from those proposed in the latter study^3^ as well as from those predicted from previous molecular dynamics simulations^4^ (Fig. 1). In addition, like those noted for SKF83959, there are subtle but notable differences of the coordination between dopamine and S198^5.42^ and S202^5.46^ compared to dopamine bound in the structures solved by the Shao group^1^ (Fig. 1). These discrepancies may be attributable to differences in structural resolution and future work will be needed to elucidate which binding pose and receptor-ligand interactions are correct, or whether the differences reflect some intrinsic dynamics of the ligands in the binding sites.

Taken together, these new cryo-EM structures of an active DRD1-Gs signaling complex provide new insights into the structural bases of agonist recognition and activation, allosteric regulation, and the selective coupling of DRD1 with Gs. Further, they provide multiple templates for the rational design of novel DRD1-selective agonists and allosteric modulators. Future work directed toward solving an inactive state structure of the DRD1 as well as the elucidation of a second known allosteric site^4^ will further exploit the therapeutic potential of this important drug target.
